# The Book World of Medicine and Science

**Published:** 1895-03-30

**Authors:** 


### The Book World of Medicine and Science.

A Manual of Ambulance. By J. Scott Riddell, C.M., M.B., M.A., Assist. Surg. Aberdeen Royal Infirmary. With numerous Illustrations and full-page Plates. (London: Charles Griffen and Co., Limited, Exeter Street, Strand. 1894.)

Chapter I. of Dr. Riddell's book consists of "Outlines of Human Anatomy and Physiology," with plates of the human skeleton complete, supplemented by other drawings of various parts of the body. The author tells us that the Manual is intended to serve not only for class use, but also as a means of reference in emergency cases. It may be feared, however, that very few members of an ambulance class will ever master Chapter I., much of which, and also some of the plates, we think might have been omitted. The chapter, however, is so good that if anyone wishes to study the subjects it treats of, he could not have a better guide. The same may be said of Chapters II. and III., which treat elaborately of bandages. The first method figured of bandaging the lower jaw should have been omitted, for the bandage would not remain *in situ* long, if fixed as directed. The second method would have been quite sufficient, as it shows the same bandage properly fixed. Chapter IV. treats of fractures, and is altogether excellent. Chapter V. is headed "Dislocations and Sprains." Only one plate is given, viz, that of a dislocation of the hip joint. Neither are the methods of reducing particular dislocations given. It is generally stated that "the treatment of a dislocation consists in reducing it—*i.e.*, in manipulating or pulling the dislocated bone into its proper relative position." In so elaborate a work we wonder directions are not found for the recognition of and the reduction of all the principal dislocations. We consider this chapter the most meagre in the book, and not worthy of insertion with the others. Chapter VI. treats of "Hæmorrhage," or bleeding, and contains a capital plate, which we do not recollect having seen elsewhere. The courses of the principal blood vessels are figured in an outline of the human body, and the exact positions where pressure should be made to arrest bleeding are indicated by arrows, so that it may be seen at a glance where pressure should be applied. The author says in collapse from bleeding alcoholic and diffusible stimulants, like tea and coffee, should be given freely if the patient can swallow, but only if the hæmorrhage has been satisfactorily arrested, and there is no probability of its reappearance on reaction setting in. This should be a first lesson, for when faintness occurs from any cause people are too prone to dose with stimulants, from which procedure injury often results. Three methods of resuscitating the apparently drowned are given in Chapter IX., viz., Sylvester's, Marshall Hall's, and Howard's, but the author truly says, "An excellent combination of the teaching of the different plans of rescue may be had on application to the secretary of the Life-saving Society." Chapter XII. treats of "Burns, Scalds, Frost-bite, and Sunstroke." It is unsatisfactory that the term "sunstroke" is retained in the nomenclature, for it is usually heated atmosphere which causes the disease, and not as the term would seem to imply a direct stroke by the rays of the sun. Most cases of so-called sunstroke occur during the night. Chapter XIV. deals with "Ambulance and Stretcher Drill," illustrated by numerous full-leaf plates. There is also a plate of a bicycle ambulance. The general rules for the proper carriage of stretchers are quoted from Mr. T. Longmore's "Treatise on Ambulance." We think, however, that such directions might be made plainer and shorter. Nor do we see that it is explicitly stated how the bearers should step. It has been found that lateral motion is prevented by the front and rear bearers starting with opposite feet. Chapter XVI. is on "The Organisation and Management of Ambulance Classes," with syllabus of lectures and questions for examination. We will quote only one question: "A young lady suddenly becomes unconscious in a tramway car. There is palor of the face and lips, and the pulse can hardly be felt. There are no other symptoms save that before becoming unconscious she complains of feeling giddy and out of sorts." It may be doubted if an adept, much less an ambulance student, would be always capable of diagnosing off-hand the cause and nature of such an attack. In conclusion, we remark that Dr. Riddell's book is an excellent one, although susceptible of improvement by condensation, omission, and addition.

